# Sleep Quality and Mental Health of Medical Students in Greece During the COVID-19 Pandemic

**DOI:** 10.3389/fpubh.2021.775374

**Published:** 2021-11-19

**Authors:** Anna Eleftheriou, Aikaterini Rokou, Aikaterini Arvaniti, Evangelia Nena, Paschalis Steiropoulos

**Affiliations:** ^1^Medical School, Democritus University of Thrace, Alexandroupolis, Greece; ^2^Department of Psychiatry, Medical School, Democritus University of Thrace, Alexandroupolis, Greece; ^3^Laboratory of Social Medicine, Medical School, Democritus University of Thrace, Alexandroupolis, Greece; ^4^Department of Pulmonology, Medical School, Democritus University of Thrace, Alexandroupolis, Greece

**Keywords:** COVID-19, medical students, sleep quality, mental health, pandemic

## Abstract

**Background-Aim:** Medical students have been greatly affected by the COVID-19 pandemic due to their educational program, which comprises theoretical knowledge and also clinical duties, making them vulnerable to viral exposures and possibly affecting their everyday life. The aim of this study was to explore changes in sleep and mental health parameters among medical students in Greece during the second year of the pandemic.

**Methods:** This cross-sectional study comprised students of all medical schools in Greece (*n* = 7), using an anonymous online survey. Participants completed the following questionnaires: Pittsburgh Sleep Quality Index (PSQI), Athens Insomnia Scale (AIS), Fatigue Severity Scale (FSS), General Anxiety Disorder-7 (GAD-7), Patient Health Questionnaire-9 (PHQ-9). Statistical analysis was conducted with the use of SPSS v.26 (IBM SPSS, Armonk NY, USA).

**Results:** Out of the 562 received responses, 559 met the inclusion criteria. The largest proportion of the respondents came from 4th-year (27.8%) and the majority of the sample were females (69.8%). Only 5.9% of the participants reported having been infected by SARS-COV-2. Most of the respondents experienced insomnia (65.9%, mean AIS score: 7.59 ± 4.24), poor sleep quality (52.4%, mean PSQI score: 6.6 ± 3.25) and increased fatigue (48.5%, mean 35.82 ± 11.74). Moderate to severe symptoms of anxiety (mean 9.04 ± 5.66) and depression (mean 9.36 ± 6.15) were noted. Suicidal ideation was found in 16.7% of the sample, while use of sleeping pills in the previous month was reported by 8.8% (*n* = 47). Further analysis revealed independent associations between sleep and mental health parameters. Higher AIS score was associated with greater FSS score; higher PSQI scores with higher GAD-7 and PHQ-9 scores. Additionally, female students were found to be significantly more affected than males by the COVID-19 pandemic, displaying higher levels of insomnia, sleep disturbances, anxiety and depression. In addition, those with a history of COVID-19 infection or in close proximity with a positive case reported significantly more significant post-traumatic symptoms in IES-COVID-19 questionnaire.

**Conclusions:** In the aftermath of the COVID-19 pandemic, prevalence of sleep and mental health disorders among Greek medical students is significant, highlighting the need for better surveillance of students' wellbeing and subsequent counseling, with special focus on female students and other affected groups.

## Introduction

The ongoing COVID-19 pandemic, which was officially declared by the World Health Organization on March 11, 2020, has caused significant changes in multiple aspects of everyday life (1, 2). Government agencies around the world have responded to this unprecedented situation by implementing measures like mandatory mask use, social distancing, travel ban and curfew, retail stores closure, contact tracing, virus detection tests and quarantine (3–5). Since the first months of the implementation of those measures, a significant impact has been described on the mental health and sleep quality of the general population (6, 7).

It was previously reported that the prolonged confinement, in combination with the growing health concerns, have resulted in a reduction in the duration and the quality of sleep of the general population. These findings were, also, positively associated with depressive symptoms (8). Similar findings were reported in Greece, with symptoms of depression and being at higher levels in certain groups, such as the younger in age (9).

A special sub-group of the population, which has been greatly affected by the above-mentioned measures, are university students. One of the first measures applied was the suspension of the operation in all educational institutions, followed by the implementation of e-learning. Also, for medical students, the clinical practice and laboratory exercise of their curriculum were paused, leading to great changes in the educational process and consequently, in their daily life (10, 11).

The new major health risk, the strict preventive measures, and the radical changes in the lifestyle of medical students are reflected on the quality of their sleep and on their mental state, as described previously (12–15). Specifically, medical students, who were concerned about the effects of COVID-19 on education and work, reported higher rates of poor sleep quality (12). Additionally, according to studies conducted during the first months of the pandemic, they presented increased rates of depression and severe anxiety, fear of stigmatization due to association with the hospital environment and anxiety of meeting the demands of the new educational reality. These findings were more likely to be more common among the female population (13–15).

However, studies conducted during the second pandemic wave, when an outburst of COVID-19 cases was reported worldwide are scarce. During that time, even stricter preventive measures were enforced, since vaccinations had not been authorized. Simultaneously, on-line education was applied for the Autumn-Winter semester of the Academic Year 2020–2021, and only medical students of the final year were allowed to resume their clinical practice. The above-mentioned developments in the course of the pandemic have caused alterations in everyday life and probably could be associated with different findings in sleep and mental health of students.

The aim of the present study was to evaluate the impact of the situation that arose during the second year of COVID-19 pandemic, on the quality of sleep and mental health i.e., anxiety and depression, of medical students in Greece.

## Materials and Methods

### Protocol and Registration

In order to enroll to the study, participants had to confirm their consent in the electronic page of the questionnaire, after being informed of the goals and the procedure of the study. Anonymity was also ensured. Prior to the initiation of the study, ethical approval was acquired (Prot. Nr. 4/22-04-2021).

### Participants

This study targeted undergraduate medical students, who completed an anonymous web-based questionnaire. The inclusion criteria were (i) currently attending one of the seven Medical Schools in Greece (ii) over 95% completion of survey questions. Answers from students pending graduation were also accepted.

### Study Design

This cross-sectional study was conducted between the 22nd of April and 31st of May 2021. During this time, members of our research team shared a post twice in several Facebook groups of students studying in the seven Medical Schools and Departments of the country, namely Aristotle University of Thessaloniki (AUTH), Democritus University of Thrace (DUTH), National and Kapodistrian University of Athens (NKUA), University of Crete (UoC), University of Ioannina (UoI), University of Patras (UPatras), and University of Thessaly (UTH). This Facebook post contained an introductory text, in which the purpose of the study was stated alongside the intention to ensure the anonymity of the participants and invited group members to participate in the study voluntarily. The post, also, provided the link to the online questionnaire, after students confirmed their consent.

### Measures

#### General Information

The initial part included questions about name of the attending University and year of studies, demographics, history of infection and hospitalization due to COVID-19.

#### Sleep Questionnaires

The Greek versions of the following validated questionnaires were included in the survey: Pittsburgh Sleep Quality Index (PSQI) (16), Athens Insomnia Scale (AIS) (17) and Fatigue Severity Scale (FSS) (18).

PSQI is a widely used self-administered questionnaire, which assesses subjectively the sleep quality of the participant over the course of the last month. PSQI measures sleep disturbances through 7 dimensions: subjective sleep quality, sleep latency, sleep duration, sleep efficiency, sleep disturbances, use of sleep medication, and daytime dysfunction. It contains 19 questions and cut-off is 5. Additional sleep disturbances can be mentioned in the relevant open-end question. Total scores range from 0 to 21 with higher scores indicating increasingly poor sleep quality (16).

AIS is a self-administered psychometric questionnaire, which assesses sleep difficulty and particularly insomnia. It contains 8 items; questions are rated on a Likert scale from 0 to 3 and total scores ≥6 indicate insomnia. Higher scores suggest severe symptoms of insomnia (17).

FSS is a self-administered questionnaire, which assesses fatigue. It contains 9 items and each one of them is scored on a 7-point Likert scale ranging from 1 to 7 (completely disagree to completely agree). Cut-off is 36 and higher scores indicate greater severity, frequency and impact of fatigue on daily life (18).

#### Mental Health Questionnaires (Symptoms of Anxiety, Depression and PTSD After COVID-19 Infection)

The participants answered the validated Greek versions of three psychometric questionnaires: General Anxiety Disorder-7 (GAD-7) (19), Patient Health Questionnaire-9 (PHQ-9) (19, 20). They also answered the Impact of Event Scale Questionnaire adapted for COVID-19 (IES-COVID19) (21, 22). Only those who had personally been infected or those who had a positive case in their familial or amical environment completed the latter.

GAD-7 is a self-administered questionnaire that assesses the severity of anxiety symptoms. It contains 7 items which are scored based on a 4-point Likert scale from 0 to 3, with higher scores indicating more severe anxiety symptoms. Cut points of 5, 10, 15 correspond to mild, moderate and severe anxiety symptoms, respectively. Scores of 10 or greater suggest a potentially clinical condition (19).

PHQ-9 is a self-administered questionnaire, which assesses depression symptoms over the course of the past 2 weeks and may be used as a tool for diagnosing clinical depression. It contains 9 items which are scored based on a 4-point Likert scale from 0 to 3, with higher scores indicating more severe depression symptoms. Cut points of 5, 10, 15, 20 correspond to mild, moderate, moderately severe and severe depression symptoms, respectively. Scores of 10 or greater suggest a potentially clinical condition (19, 20).

IES-COVID19 is a 15-item self-administered questionnaire, which is designed to assess subjective distress during the past 7 days over experiencing a COVID-19 infection either personally or of the immediate environment. Every item is rated on a 4-point scale (0: not at all, 1: seldom, 3: sometimes and 5: often). Higher scores indicate a higher psychological impact of the COVID-19 infection (21, 22). The IES-COVID 19 could be used in a preventive manner by screening individuals at high risk for developing PTSD.

### Statistical Analysis

Statistical analysis was conducted with the use of SPSS v.26 (IBM SPSS, Armonk NY, USA). Normality of distribution was checked with Kolmogorov-Smirnov test and appropriate analyses were applied. Descriptive and analytic statistics were used. Statistical significance was set at *p* < 0.05 level.

## Results

### General Characteristics

In total, 562 answers were received, out of which, 559 were eligible for analysis (completion rate 99.46%). Table 1 displays the characteristics of the participants. As seen, all Universities were represented, with variable participation rates. The majority were females, with a larger participation coming from students of the 4th year. Thirty-three (5.9%) respondents have been infected by SARS-COV-2 and 215 (38.4%) reported at least one positive case of COVID-19 in their approximate environment.

**Table 1:** General characteristics of the participants.

	**Number** ** (N)**	**Percentage** ** (%)**
**Participants (valid answers)**	559	
**Sex (M/F)**	164/389	
**Medical university**		
AUTH	79	14.2
DUTH	117	21
NKUA	107	19.2
UoC	95	17
UoI	45	8.1
UPatras	66	11.8
UTH	49	8.8
**Academic year**		
1st	71	12.7
2nd	71	12.7
3rd	78	14
4th	155	27.8
5th	81	14.5
6th	80	14.3
Pending graduation	22	3.9
**COVID-19 infection**		
No infection	328	58.7
Only personally infected	16	2.9
Infection only in members of the close environment	198	35.2
Infection both personally and in the close environment	17	3

*AUTH, Aristotle University of Thessaloniki (Thessaloniki, Greece); DUTH, Democritus University of Thrace (Alexandroupolis, Greece); NKUA, National and Kapodistrian University of Athens (Athens, Greece); UoC, University of Crete (Heraklion, Greece); UoI, University of Ioannina (Ioannina, Greece); UPatras, University of Patras (Patras, Greece); UTH, University of Thessaly (Larissa, Greece)*.

### COVID-19 Pandemic, Sleep Quality and Sleep Characteristics

The second part of the survey assessed the sleep quality of the participants spanning over a period of 1 week −1 month before its completion. Most of the respondents (*n* = 368, 66%) experienced insomnia, according to their AIS score (mean 7.59 ± 4.24), with females being significantly more affected (mean AIS female score: 7.88 ± 4.2, mean AIS male score: 6.88 ± 4.4, *p* = 0.012).

More than half of participants (*n* = 293, 52.4%) evaluated their sleep as being of poor quality, according to PSQI score. Sleep disturbances were reported by almost all participants (499/559 participants), including fragmented sleep, snoring, difficulty in breathing and nightmares. Additional sleep disturbances, as mentioned in the relevant open-end question, were anxiety, stress, and loneliness as demonstrated in more detail in Table 2. Interestingly, 8.8% (*n* = 47) of university students stated that they have used sleep- promoting medication during the past month. Almost half of the participants reported increased levels of fatigue (*n* = 270, 48.5%, mean: 35.82 ± 11.74).

**Table 2:** Reported sleep disturbances (open-end answers only).

**Sleep disturbances**	**Number of participants**
Stress–Anxiety^*^	30
Negative thoughts–overthinking	13
Use of technology	5
Palpitations	5
Tension	5
Fear	3
Loneliness	3
Headaches	2
Anger	2
Melancholy–sadness	2
Panic attack	1
Sleep paralysis	1

* *About possible COVID-19 infection, increased workload, examinations*.

Comparisons between students of different academic years revealed statistically significant variations in the duration of their sleep (i.e., third PSQI component). Specifically, students of the 6th year reported significantly shorter sleep duration (*p* = 0.003).

### COVID-19 Pandemic and Mental Distress

The third part of the survey addressed the effects of the COVID-19 pandemic on different aspects of mental health. The majority of participants (*n* = 377, 67.6%) reported moderate to severe symptoms of anxiety (mean 9.04 ± 5.66). More specifically, 28.4% reported moderate symptoms, 23.1% moderately severe symptoms and 16.2% severe symptoms.

Similar results regarding depression symptoms were found (mean score: 9.36 ± 6.15), with 22.6% having moderate depression symptoms, 13.9% moderately severe depression symptoms, and 7.2% severe depression symptoms. Mild depression symptoms were reported in 30.6% of the respondents. Notably, a non-neglectable percentage (*n* = 92, 16.7%) of the participants, regardless of gender (*p* = 0.579), reported being affected by recurrent suicidal thoughts (several days: 9.8%, More than half of the days: 3.3%, nearly every day: 3.6%). Comparison analysis between COVID-19 infection status and depression levels (as indicated by PHQ-9) showed that students who had both themselves and their immediate environment infected, experienced symptoms in a more severe way (not infected: 9.03 ± 6.3 vs. only immediate environment infected: 9.89 ± 5.54, infected both themselves and their immediate environment: 12.29 ± 8.89; *p* = 0.022).

Comparison between genders revealed that females were experiencing significantly more severe symptoms in all mental health measures scores (GAD-7 mean score females: 9.4 ± 5.66, males: 8.16 ± 5.64, *p* = 0.02, PHQ-9 mean score females: 9.95 ± 6.16, males: 7.93 ± 5.98, *p* = 0.001, IES-COVID19 mean score females: 25.50 ± 13.53, males: 20.69 ± 12.21, *p* = 0.018).

Additionally, female students infected by COVID-19 or in close proximity with a positive case of the disease, reported significantly more frequently bad dreams (*p* = 0.025), and persistent negative thoughts or images (*p* = 0.031, *p* = 0.048, respectively) according to IES-COVID19 scores.

### Correlations Between Sleep and Mental Parameters

As a next step, a correlation analysis between sleep and mental health parameters was conducted, revealing numerous independent associations among them as shown in Table 3. There was a statistically significant correlation between all scales. Higher levels of insomnia (according to the AIS score) were associated with greater severity and frequency of fatigue (as described by the FSS score); poor quality of sleep (resulting from PSQI) was associated with higher levels of anxiety and depression symptoms (as indicated by the GAD-7 and PHQ-9 scores).

**Table 3:** Correlations between sleep and mental parameters.

		**AIS score**	**FSS score**	**PSQI score**	**GAD-7 score**	**PHQ-9 score**
AIS Score	Pearson correlation coefficient (r)	1	0.496	0.684	0.556	0.633
	*p*		<0.001	<0.001	<0.001	<0.001
FSS score	Pearson correlation coefficient (r)	0.496	1	0.426	0.506	0.609
	*p*	<0.001		<0.001	<0.001	<0.001
PSQI score	Pearson correlation coefficient (r)	0.684	0.42	1	0.487	0.566
	*p*	<0.001	<0.001		<0.001	<0.001
GAD-7 score	Pearson correlation coefficient (r)	0.556	0.506	0.487	1	0.704
	*p*	<0.001	<0.001	<0.001		0.000
PHQ-9 score	Pearson correlation coefficient (r)	0.633	0.609	0.566	0.704	1
	*p*	<0.001	<0.001	<0.001	<0.001	

*AIS, Athens Insomnia Scale; FSS, Fatigue Severity Scale; PSQI, Pittsburgh Sleep Quality Index; GAD-7, General Anxiety Disorder-7; PHQ-9, Patient Health Questionnaire-9*.

## Discussion

Our study captures the alterations in sleep quality and mental health of medical students in Greece during the second year of the COVID-19 pandemic. Overall, regarding sleep parameters, the respondents reported impaired sleep quality due to multiple sleep disturbances and decreased sleep duration; this was more obvious among 6th year medical students. Higher levels of insomnia, especially in females and increased fatigue in daily life were also reported. These alterations in sleep parameters were correlated with moderate to severe deterioration of mental health. Respondents presented also moderate to severe symptoms of anxiety and depression, to the point that a significant proportion admitted recurrent suicidal thoughts.

So far, contradictory findings are available in the literature regarding alterations in students' sleep schedule and sleep quality and the majority refers to the first year of the COVID-19 pandemic until the end of December 2020. A recent study conducted in 7 countries showed a prevalence of poor sleep among students worldwide and deficient sleep duration in more than one out of four students (23). These findings are in accordance with other studies that describe reduced night sleep duration and sleep efficiency due to sleep disturbances (24–26). Analogous conclusions have been drawn specifically for medical students and have been linked to disturbed daytime function (27). Notably, the senior medical students experienced significantly more intensely these alterations (12). However, in other studies insignificant changes in sleep quality (28) and an overall improvement in daily performance have been reported (29). Increased daytime napping though seems to offset sleep latency, thus total sleep duration remained unaffected (24). Increased total sleep time has also been mentioned, compared to the pre-pandemic period, especially among 6th year medical students (29). In our study, however, this specific population group reported the lowest sleep duration compared to students from other academic years. This can be possibly attributed to the resumption of their clinical practice and the clinical responsibilities during the pandemic, which may be linked with fear and anxiety, as previously shown (30).

Besides sleep schedule, during the COVID-19 health crisis, the mental health of students has been significantly affected. In our study 67.6 and 43.7% of the sample reported moderate to severe symptoms of anxiety and depression respectively. According to a recent systematic review by Batra et al. (31) performed in 15 countries, anxiety and depression levels reached 39.4 and 31.2% among university students, respectively. This study was conducted almost 1 year before our study, and thus during this period the psychological distress and depression have accumulated, possibly explaining the difference in our results (31). Interestingly, in two of the studies included in this systematic review almost one out of three and two out of three students, respectively, had suicidal ideation, which is surprisingly high compared to our results (32, 33). In our study, 16.7% of the respondents suffered from recurrent suicidal thoughts, which is in accordance with previous findings (34, 35). Furthermore, during the pandemic period, a rise in depression has been noted, with its severity and prevalence varying between different studies (12, 36–38). Additionally, the prevalence of PTSD symptoms, in those studies, was similarly elevated (31), with a higher degree of anxiety being attributed to increased concern about the impact of the COVID-19 pandemic (39) and the presence of a confirmed COVID-19 case in the proximal familial and friendly environment (40). This is also the case among medical students (36, 41), who experienced analogous levels of stress and anxiety symptoms (36, 37). According to our findings, infection in the immediate environment is associated with depression in a more severe way and PTSD symptoms, mainly in females.

Published literature associating gender and mental health is inconclusive, for example in the study by Xie et al. (12), males reported depressive symptoms more frequently, whereas Liu et al. found no statistically significant difference between gender with regards to anxiety and depression (37). However, Batra et al. (31) in their systematic review found that female students experienced higher levels of anxiety and stress. These results are consistent with our study. An interesting finding was that females having experienced COVID-19 either personally or in their proximal environment were significantly more affected than their male counterparts. A possible explanation could be that women in general, are more likely to report experiencing higher levels of anxiety (42) and that they are more affected by traumatic events (31).

Another finding in our study was the positive and independent correlation between insomnia, fatigue, dysfunctional sleep, depressive and anxiety symptoms. Previous studies confirm the association between sleep abnormalities and deteriorated mental health in students during the COVID-19 health crisis (26, 36). This comes as no surprise, since similar correlations have already been established, even before the pandemic. In a cross-sectional study of 95 medical students in Saudi Arabia stress, anxiety and depression were strongly linked with poor sleep (43). It was also reported that inadequate sleep duration and consequently fatigue may affect mental health to such a degree, that recurrent suicidal thoughts and even suicidal attempts may occur more frequently (44). Additionally, it has been demonstrated, both in the general population and specifically in medical students, that insomnia can be predictive of depression and anxiety (45, 46).

In a large study of the general population in Greece, Switzerland, Austria, Germany, France and Brazil, conducted during the first wave of the pandemic, total sleep time decreased and sleep quality in general improved in participants from Greece, compared to other countries (47). At the same time, insomnia affected 37.6% in a sample of the Greek population, which was significantly increased compared to the pre-pandemic period (48). As far as mental health is concerned, levels of anxiety and depression were notably elevated during the first COVID-19 wave. Fountoulakis et al. reported a significant increase in anxiety symptoms in over 45% and depressive symptoms in almost 40% of the participating Greek citizens (49). According to Patsali et al. major depression in the general population reached 12.43% (50). Focusing on Greek students, during the first pandemic wave, they experienced overall lower sleep quality despite an increase in their sleep duration (33). Our findings suggest an even higher prevalence of insomnia in our selected population (medical students) compared to the general population, affecting 65.9% of the participants. Kaparounaki et al. noted anxiety in 73%, depression in 60.9% and suicidal ideation in 20.2% in a Greek university sample (33). Meanwhile in a study conducted in the University of Patras by Sazakli et al., anxiety symptoms during COVID-19 pandemic decreased to 35.8% and depressive symptoms increased to 51.2% (51). Interestingly, in our study, anxiety levels were significantly higher and reached 67.6%. This also the case with our reported levels of depression, where overall 74.3% of the participants experienced it to some degree and 43.7% admitted having moderate to severe symptoms.

Our study certainly has limitations. Firstly, participation rate was relatively low; however, it is representative of the experiences of medical students since respondents came from all Greek Medical Schools. Additionally, examined parameters were assessed with the use of self-administered questionnaires in an on-line survey. On the other hand, we have used a large number of diagnostic tools, validated for the Greek population, and already used in several studies and thus are ensuring standardized results. In addition, this is, to the best of our knowledge, the first study to assess the impact of COVID-19 pandemic on sleep parameters of medical students in Greece, in association with a large series of sleep and mental health parameters.

## Conclusions

In the aftermath of the COVID-19 pandemic, Greek medical students experienced, in a greater degree, sleep and mental health disorders such as insomnia, fatigue, poor sleep quality, anxiety, post-traumatic stress and depression. Thus, the need for better surveillance of students' wellbeing and subsequent counseling is even more evident now. A special focus must be given to the most affected groups such as female students.

## Data Availability Statement

The raw data supporting the conclusions of this article will be made available by the authors, upon request.

## Ethics Statement

The study protocol, which involved human participants, was reviewed and approved by University General Hospital of Alexadroupolis Scientific Board. The patients/participants provided their informed consent to participate in this study.

## Author Contributions

AE, AR, and PS designed the study and wrote the manuscript. AA and EN contributed to the interpretation of the results and provided critical feedback. All authors contributed to the article and approved the submitted version.

## Conflict of Interest

The authors declare that the research was conducted in the absence of any commercial or financial relationships that could be construed as a potential conflict of interest.

## Publisher's Note

All claims expressed in this article are solely those of the authors and do not necessarily represent those of their affiliated organizations, or those of the publisher, the editors and the reviewers. Any product that may be evaluated in this article, or claim that may be made by its manufacturer, is not guaranteed or endorsed by the publisher.
